# Supplementary material for: Impact of Salvage Surgery following Colonic Endoscopic Polypectomy for Patients with Invasive Neoplasia
Source: Curr Oncol. 2022 Apr 29;29(5):3138–48. doi: 10.3390/curroncol29050255 (PMC9139913; doi:10.3390/curroncol29050255)
Supplement: Supplementary file 1 [file curroncol-29-00255-s001.zip › curroncol-1659983-supplementary.pdf]

Current Oncology

MDPI

Article

Impact of Salvage Surgery Following Colonic Endoscopic Polypectomy for Patients with Invasive Neoplasia

Xiangzhou Tan, Markus Quante, Zihua Chen, Zhikang Chen, Alfred Königsrainer and Dörte Wichmann

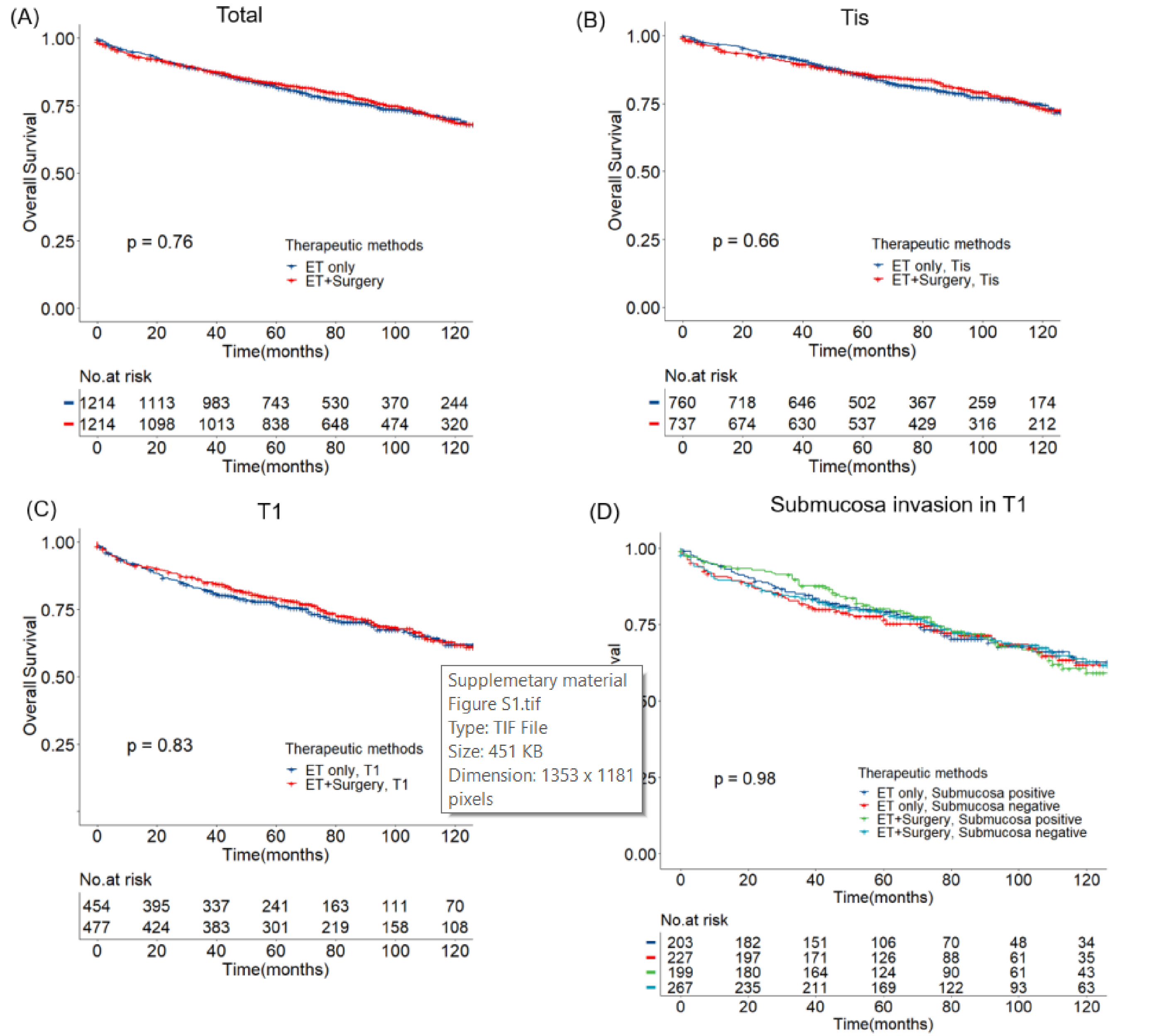

Figure S1. Kaplan-Meier overall survival plots in patients underwent endoscopic removal of invasive neoplasia. (A) All patients; (B) Patients with intramucosal carcinoma (Tis); (C) Patients with submucosal carcinoma (T1). (D) Patients with or without submucosa invasion.

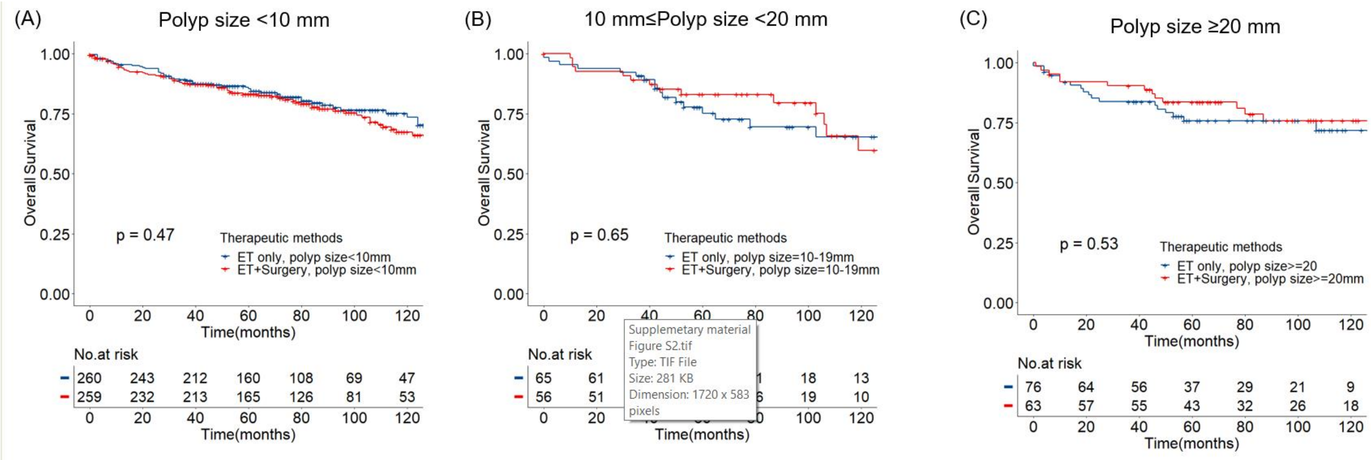

Figure S2. Kaplan-Meier overall survival plots in patients underwent endoscopic removal of invasive neo-plasia. (A) Invasive polyp size less than 10 mm; (B) Invasive polyp size between 10 mm and 20 mm; (C) Invasive polyp size larger than 20 mm.
